# *ECHS1*: pathogenic mechanisms, experimental models, and emerging therapeutic strategies

**DOI:** 10.1186/s13023-025-03959-y

**Published:** 2025-08-13

**Authors:** Qiang Fu, Rui Qiu, Shang Li, Yuxiang Qin, Ziyi Lu, Shanxin Liyao, Zimo Yang, Xiang Cheng, Yuewen Chen, Huan Xu, Yong Cheng

**Affiliations:** 1 Center on Translational Neuroscience, Institute of National Security, Minzu University of China, 27th South Zhongguancun Avenue, Beijing, 100081 China; 2 School of Ethnology and Sociology, Minzu University of China, Beijing, China; 3 College of Life and Environmental Sciences, Minzu University of China, Beijing, China; 4 Chinese Academy of Sciences Key Laboratory of Brain Connectome and Manipulation, Shenzhen Key Laboratory of Translational Research for Brain Diseases, The Brain Cognition and Brain Disease Institute, Shenzhen Institute of Advanced Technology, Chinese Academy of Sciences, Shenzhen – Hong Kong Institute of Brain Science – Shenzhen Fundamental Research Institutions, Shenzhen, 518055 Guangdong China; 5 Guangdong Provincial Key Laboratory of Brain Science, Disease and Drug Development, HKUST Shenzhen Research Institute, Shenzhen, 518057 Guangdong China; 6 Department of Clinical Laboratory, General Hospital of Xizang Military Command, Lhasa, 850000 China

**Keywords:** *ECHS1* protein, human, Mitochondrial diseases, Fatty acids, volatile, Beta-oxidation, Genetic therapy

## Abstract

The *ECHS1* (short-chain enoyl-CoA hydratase 1) gene is critical for mitochondrial fatty acid β-oxidation and branched-chain amino acid metabolism. Mutations in *ECHS1* lead to severe mitochondrial dysfunction and are implicated in rare metabolic and neurodegenerative disorders. This review summarizes current understanding of how *ECHS1* participates in key molecular processes, including energy metabolism, oxidative stress regulation, and apoptosis, and discusses its influence on mitochondrial function. It also highlights advances in experimental models, including mouse, Drosophila, and induced pluripotent stem cell (iPSC) -based systems, which have illuminated the gene’s physiological roles while revealing model-specific limitations. Therapeutic approaches, such as dietary interventions, gene therapy, enzyme replacement therapy, and stem cell therapy, are critically evaluated, emphasizing their potential and current challenges. Despite significant progress, gaps remain in understanding *ECHS1*’s tissue-specific and developmental-stage-specific functions. This review underscores the need for advanced human-relevant models and integrative technologies to address these gaps and foster the development of personalized treatments for *ECHS1*-related disorders.

## *ECHS1* discovery and clinical features

The *ECHS1* gene, located on chromosome 10q26.3, spans approximately 41.3 kb and contains eight exons. It encodes short-chain enoyl-CoA hydratase 1, a mitochondrial matrix enzyme that plays a critical role in fatty acid β-oxidation and branched-chain amino acid metabolism [1]. Early biochemical studies established its function in cellular energy metabolism, laying the foundation for understanding its broader physiological relevance. Subsequent research identified mutations in the *ECHS1* gene as a causative factor in various mitochondrial disorders, most notably Leigh syndrome. This condition is clinically characterized by neurodevelopmental regression, bilateral basal ganglia lesions, lactic acidosis, and early-onset encephalopathy, all of which reflect the central role of *ECHS1* in maintaining mitochondrial energy homeostasis [2–4].

With advances in molecular biology, functional genomics, and patient-derived disease models, the pathophysiological roles of *ECHS1* have been further clarified [5]. In particular, enzymatic assays and molecular characterization from the late 20th to early 21st century confirmed that ECHS1 catalyzes the hydration of short-chain enoyl-CoA, a key step in mitochondrial β-oxidation, essential for metabolic stability.

The widespread adoption of genome sequencing technologies has led to the identification of numerous disease-associated *ECHS1* mutations, which typically result in reduced or absent enzyme activity. This enzymatic dysfunction disrupts energy metabolism, particularly in high-demand tissues such as the brain, leading to severe neurological symptoms. As a result, research focus has increasingly shifted from basic biochemical characterization toward clinical investigation and therapeutic development for *ECHS1*-related mitochondrial disorders.

### Clinical characteristics

Clinically, diseases associated with *ECHS1* mutations exhibit high phenotypic heterogeneity. Symptoms are largely determined by the mutation type, localization, and extent of enzymatic impairment. Most patients with *ECHS1* mutations present with significant neurological symptoms in the neonatal or early infancy period [6–8]. Common manifestations include hypotonia, delayed motor development, seizures, and dystonia, indicating widespread neurological involvement [9, 10]. These symptoms tend to worsen as the disease progresses, resulting in further regression of motor and cognitive development [11]. In addition to neurological symptoms, patients with *ECHS1* mutations often display notable metabolic abnormalities, with elevated lactate and pyruvate levels in blood and cerebrospinal fluid, suggesting severe mitochondrial dysfunction—a hallmark of metabolic diseases [12, 13].

MRI is a crucial diagnostic tool for detecting *ECHS1* mutation-related diseases. Typically, MRI findings reveal bilateral T2 hyperintensity in the basal ganglia, a characteristic indicator of metabolic encephalopathy [14]. In some patients, MRI also shows extensive white matter damage and thinning of the corpus callosum, suggestive of neurodegenerative changes [15]. These imaging characteristics correlate with the neurological symptoms and provide reliable diagnostic evidence.

**Fig. 1:**
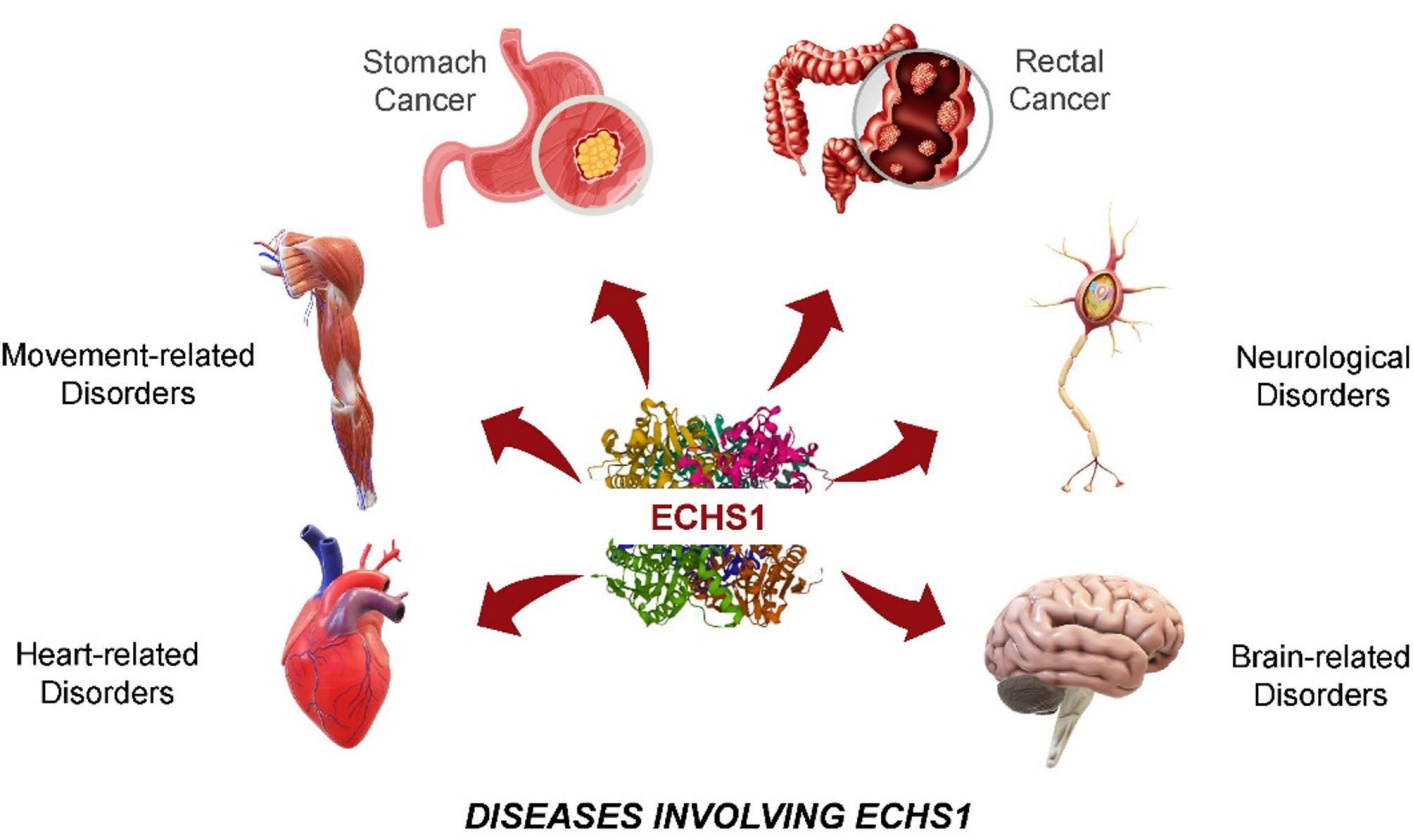
*ECHS1* dysfunction contributes to multisystem disorders via disrupted mitochondrial metabolism. This schematic illustrates the pathological impact of *ECHS1* gene mutations, represented by its encoded protein at the center, and their association with a range of clinically distinct disorders. Loss of short-chain enoyl-CoA hydratase activity leads to impaired valine catabolism and fatty acid β-oxidation, resulting in mitochondrial dysfunction and widespread metabolic imbalance. Mechanistic links (indicated by red arrows) connect *ECHS1* deficiency with increased cancer susceptibility—particularly gastric adenocarcinoma and colorectal carcinoma—due to accumulation of oncometabolites. Neuromotor disorders, such as dystonia and chorea, as well as cardiomyopathy stemming from mitochondrial energy failure, are also observed. In addition, *ECHS1* mutations underlie complex neurological symptoms, including developmental delay, seizures, and encephalopathy, and contribute to structural brain abnormalities like those seen in Leigh-like syndrome with basal ganglia lesions. These overlapping phenotypes reflect the systemic consequences of mitochondrial metabolic disruption caused by *ECHS1* deficiency

Moreover, the systemic effects of *ECHS1* mutations are not limited to the nervous system; some patients also exhibit cardiomyopathy, optic atrophy, movement disorders, and other systemic symptoms, with potential associations even reported with gastric and rectal cancers [16](Fig. 1). This multisystem involvement not only complicates the diagnosis but also suggests the gene’s complex role in metabolic diseases.

### Mutation types and clinical significance

To date, reported *ECHS1* mutations include missense, nonsense, splice-site, and frameshift mutations. The impact of these mutations on protein function varies, resulting in diverse clinical phenotypes. For instance, compound heterozygous mutations such as c.796 A > G (p.T266A) and c.463G > A (p.G155S) have been widely documented in the literature and are frequently associated with severe mitochondrial dysfunction and neurological deficits [17, 18]. Recent studies have identified novel mutations, including c.414 + 5G > A and c.310 C > G, which significantly alter protein structure and reduce enzymatic activity, leading to severe metabolic disease [11, 17]. Systematic aggregation and analysis of known mutation sites help elucidate the effects of different mutations on ECHS1 function, providing a theoretical basis for investigating its pathological mechanisms.

### Current clinical research and limitations

Despite significant progress in the clinical research of *ECHS1* mutations over recent years, several limitations remain. While associations between *ECHS1* mutations, metabolic abnormalities, and neurological impairment are recognized, the specific molecular mechanisms and signaling pathways remain largely unexplored. This lack of mechanistic understanding limits our insights into the disease’s pathogenesis. Although *ECHS1* gene mutations are challenging to screen for prenatally, the metabolic disturbances they cause can often be detected through abnormal metabolites in patient urine. By summarizing the abnormal urinary metabolites associated with *ECHS1* mutations, we aim to contribute to potential screening tools that may enable earlier diagnosis and timely intervention (Table 1). To provide a more comprehensive understanding of the metabolic context in which ECHS1 functions, detailed information on the valine, leucine, and isoleucine degradation pathway is available from the NCBI Bookshelf (https://www.ncbi.nlm.nih.gov/books/NBK542806/) and the KEGG Pathway Database (https://www.kegg.jp/entry/hsa:1892), which together offer valuable insights into the biochemical framework surrounding ECHS1 activity.

**Table 1:** Potential abnormal urinary metabolites in ECHS1 deficiency

Category	Metabolites	Metabolic Pathway
Methacrylate Metabolites	S-(2-carboxypropyl)cysteine, S-(2-carboxypropyl)cysteamine	Valine Pathway
Acrylate Metabolites	S-(2-carboxyethyl)cysteine, S-(2-carboxyethyl)cysteamine	Valine Pathway
Crotonate Metabolites	Crotonyl glycine	Isoleucine Pathway
Butyrate Metabolites	Crotonyl glycine	Fatty Acid Pathway
Other Branched-Chain Amino Acid Metabolites	2-Methyl-2,3-dihydroxybutyric acid, 3-hydroxyisovaleric acid, pyruvic acid, 3-methylglutaconic acid, ketone bodies, lactic acid	General Branched-Chain Amino Acid Metabolites
Derived Urinary Metabolites	N-acetyl-methacryl-L-cysteamine, acryl-cysteamine, acryl-L-cysteamine, N-acetyl-acryl-cysteine, methacryl-cysteamine	Urinary Metabolites Related to Multisystem Metabolism

Current treatments for ECHS1-related metabolic disorders primarily focus on symptomatic support, such as seizure management and metabolic stabilization. With advancements in gene editing and drug screening, personalized therapies may become feasible in the future. Potential strategies include gene editing to correct mutations or screening for targeted small molecules to restore mitochondrial function. These approaches could significantly improve patient outcomes and provide new therapeutic directions for other inherited metabolic disorders.

## ECHS1 molecular mechanisms

### ECHS1 in fatty acid β-oxidation

ECHS1 plays a critical role in the mitochondrial β-oxidation of fatty acids, a process essential for breaking down long-chain fatty acids into acetyl-CoA [19]. Acetyl-CoA subsequently enters the tricarboxylic acid (TCA) cycle, thereby providing cellular energy [20]. ECHS1 facilitates the hydration step in β-oxidation by converting 2-enoyl-CoA to 3-hydroxyacyl-CoA, a reaction crucial for the progression of fatty acid breakdown [21].

**Fig. 2:**
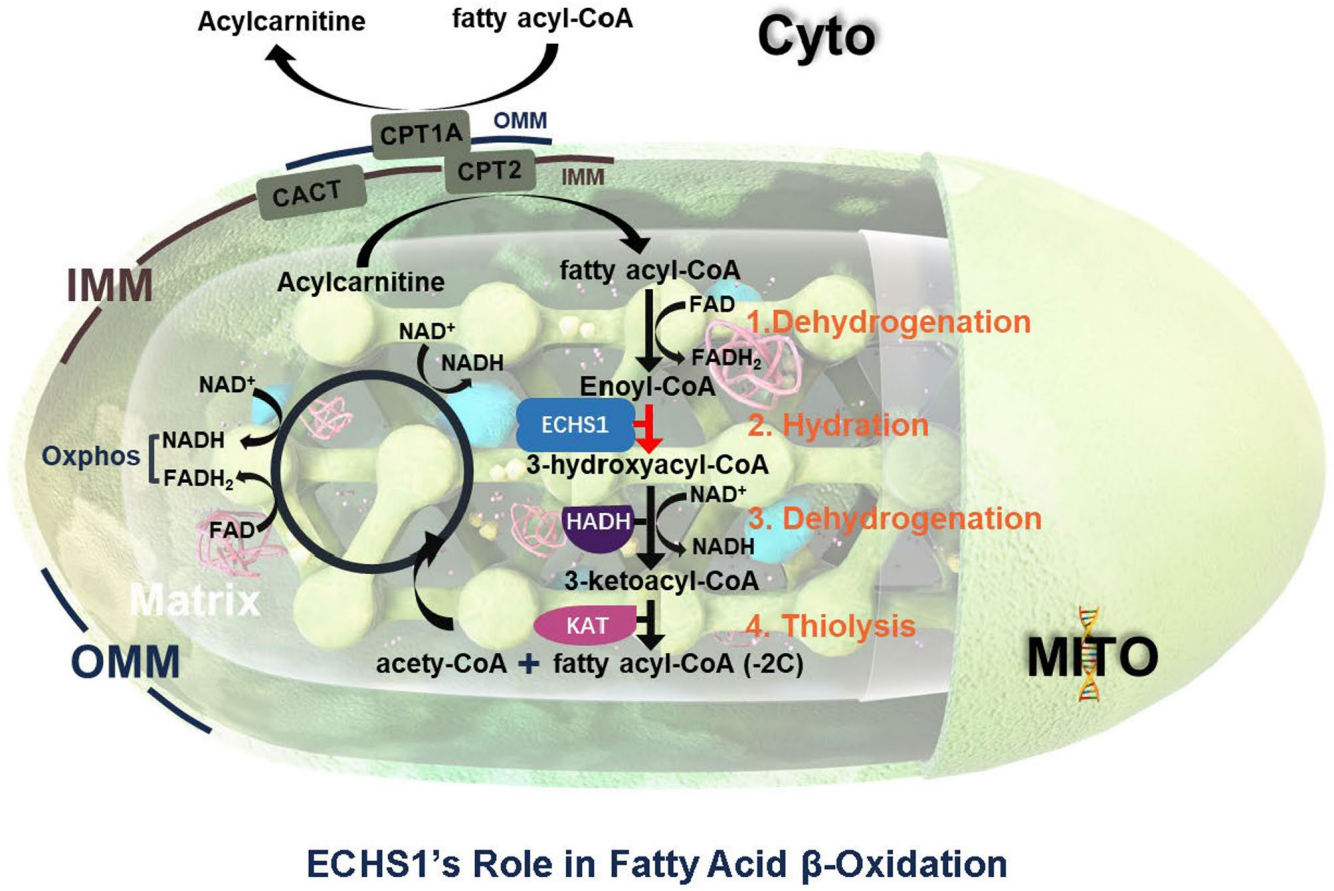
ECHS1’s role in fatty acid β-oxidation. This diagram illustrates the four key enzymatic steps involved in mitochondrial fatty acid β-oxidation, emphasizing the role of *ECHS1* in the second step—hydration of enoyl-CoA to 3-hydroxyacyl-CoA. Following transport of fatty acyl-CoA into the mitochondrial matrix via the carnitine shuttle system (CPT1A, CACT, CPT2), sequential reactions—dehydrogenation (FAD-dependent), hydration (*ECHS1*-mediated), NAD⁺-dependent dehydrogenation, and thiolysis—produce acetyl-CoA and a shortened acyl-CoA chain. *ECHS1* deficiency disrupts this pathway, impairing mitochondrial energy production and contributing to diseases such as Leigh syndrome. (Cyto: Cytoplasm; MITO: Mitochondrial ; OMM: Outer Mitochondrial Membrane; IMM: Inner Mitochondrial Membrane; Matrix: Mitochondrial Matrix; CPT1A: Carnitine Palmitoyltransferase 1 A; CACT: carnitine acylcarnitine translocase; CPT2: carnitine O-palmitoyltransferase 2; FAD: Flavin Adenine Dinucleotide; FADH₂: Reduced Flavin Adenine Dinucleotide; ECHS1: Enoyl-CoA Hydratase, Short-chain 1; HADH: Hydroxyacyl-CoA Dehydrogenase; NADH: Reduced Nicotinamide Adenine Dinucleotide; KAT: Ketoacyl-CoA Thiolase; NAD⁺: Nicotinamide Adenine Dinucleotide (oxidized form))

During the four-step β-oxidation cycle, ECHS1 catalyzes the second step, hydrating 2-enoyl-CoA to form 3-hydroxyacyl-CoA [22]. This step directly regulates the turnover of fatty acid metabolites and ensures a steady production of acetyl-CoA for cellular energy [23]. The active site of ECHS1 binds precisely to 2-enoyl-CoA, performing a catalytic function particularly critical in energy-demanding tissues such as cardiac and skeletal muscle [24](Fig. 2).

Deficiency or mutation in ECHS1 disrupts the β-oxidation cycle, leading to metabolic consequences. Blockage of the hydration step causes accumulation of 2-enoyl-CoA and other intermediates, which can be toxic to cells and may interfere with other metabolic pathways. Additionally, decreased acetyl-CoA supply limits the TCA cycle’s capacity to generate ATP, especially under energy-demanding conditions like fasting [25]. Disruption in fatty acid breakdown also elevates mitochondrial oxidative stress, with excess intermediates generating reactive oxygen species (ROS) that damage cellular structures [26]. When β-oxidation is impaired, cells rely more heavily on glycolysis, resulting in lactate buildup and lactic acidosis, which exacerbates metabolic imbalance.

In summary, ECHS1’s catalytic role in fatty acid β-oxidation is essential for maintaining normal cellular metabolism. Deficiency or mutation in ECHS1 leads to the accumulation of fatty acids and metabolic intermediates, increased oxidative stress, reduced energy production, and metabolic acidosis. Understanding this mechanism provides critical insights into the pathogenesis of ECHS1-related metabolic diseases and offers a foundation for developing potential therapeutic strategies.

### ECHS1’s role in branched-chain amino acid metabolism

**Fig. 3:**
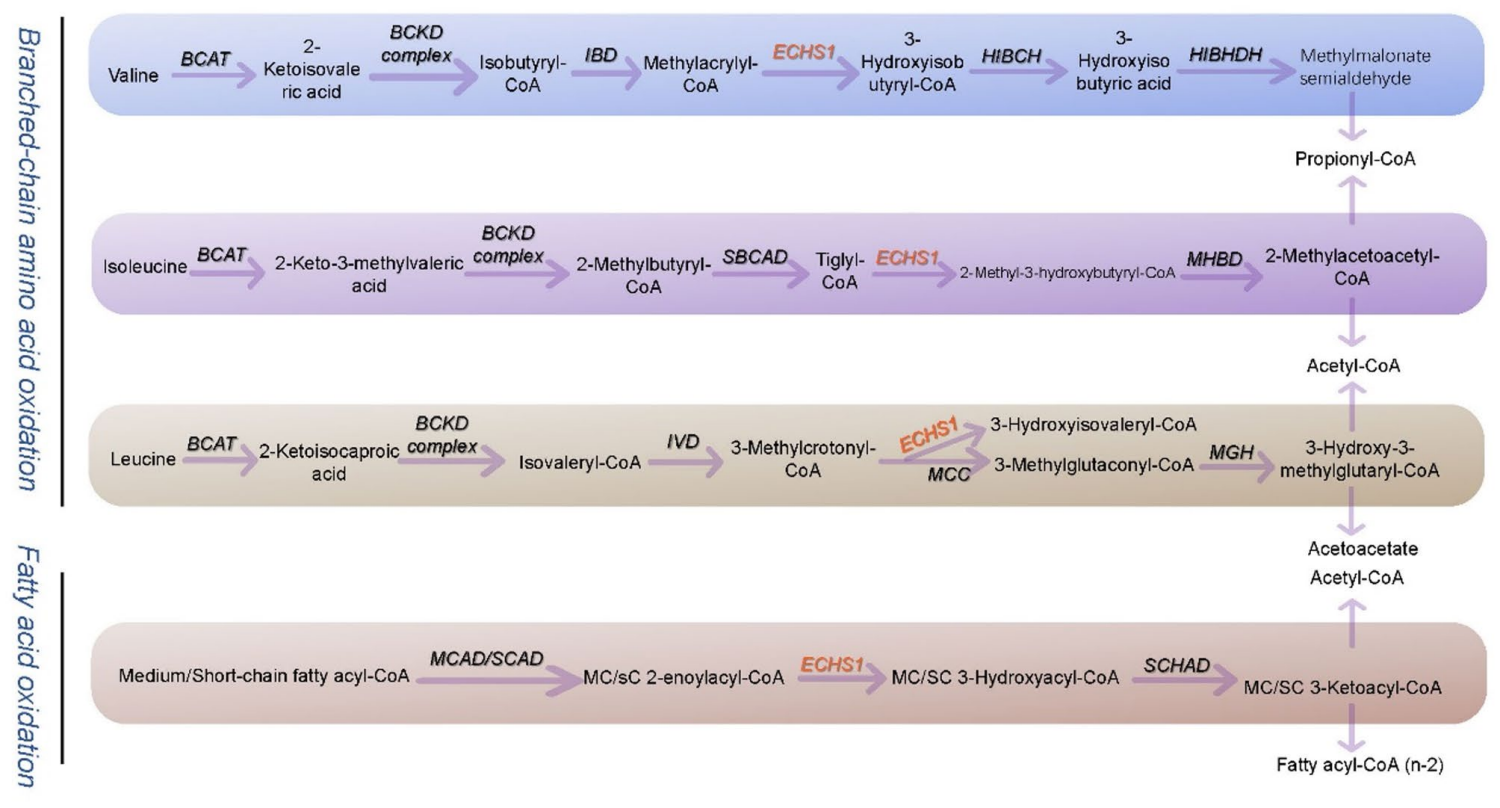
Mechanism of *ECHS1* in branched-chain amino acid metabolism. This schematic illustrates the dual enzymatic function of short-chain *ECHS1* in mitochondrial energy metabolism. The blue pathway represents the degradation of branched-chain amino acids (valine, isoleucine, and leucine), while the beige pathway outlines fatty acid β-oxidation. *ECHS1* catalyzes the hydration of enoyl-CoA intermediates derived either from branched-chain amino acids—via the BCKD complex—or from fatty acids—via SCAD/SBCAD. The downstream conversion of these intermediates by SCHAD and other mitochondrial enzymes leads to the generation of acetyl-CoA and propionyl-CoA. Metabolic cross-talk is highlighted at key nodes, including methylmalonate semialdehyde and methylglyoxal. Enzyme abbreviations include BCAT, IVD, MCC, and HMG-CoA lyase, demonstrating *ECHS1*’s central role in maintaining mitochondrial metabolic flux through its dual substrate specificity. (BCAT: branched-chain aminotransferase; BCKD complex: branched-chain alpha-keto acid dehydrogenase complex; HIBCH: 3-hydroxyisobutyryl-CoA hydrolase; HIBADH: 3-hydroxyisobutyrate dehydrogenase; HMG-CoA lyase: 3-hydroxy-3-methylglutaryl-CoA lyase; MHBD: 2-methyl-3-hydroxybutyryl-CoA dehydrogenase; MGH: 3-methylglutaconyl-CoA hydratase; IBD: isobutyryl-CoA dehydrogenase; IVD: isovaleryl-CoA dehydrogenase; MCAD: medium-chain acyl-CoA dehydrogenase; MCC: 3-methylcrotonyl-CoA carboxylase; MMSDH: methylmalonate semialdehyde dehydrogenase; SBCAD: short branched-chain acyl-CoA dehydrogenase; SCAD: short-chain acyl-CoA dehydrogenase; SCEH: short-chain enoyl-CoA hydratase; SCHAD: short-chain 3-hydroxyacyl-CoA dehydrogenase.)

ECHS1 is a critical enzyme in the metabolism of branched-chain amino acids (BCAAs), particularly valine. BCAAs serve dual roles in cellular metabolism: they provide essential energy (ATP) to mitochondria and generate intermediates that support antioxidant defense mechanisms and intracellular signaling pathways [27]. Thus, maintaining BCAA metabolic balance is indispensable for cellular homeostasis. A pivotal step in BCAA degradation involves the decarboxylation of valine to produce isobutyryl-CoA, which subsequently enters the β-oxidation pathway [28]. Within this pathway, ECHS1 catalyzes the hydration of 2-enoyl-CoA to 3-hydroxyacyl-CoA, a critical reaction that facilitates the generation of acetyl-CoA and propionyl-CoA, key substrates for mitochondrial energy production and metabolic equilibrium (Fig. 3) [29].

Disruptions in ECHS1 function result in the pathological accumulation of branched-chain α-keto acids and their intermediate metabolites, which cannot be efficiently degraded [30]. These toxic intermediates exhibit pronounced neurotoxicity, particularly in neuronal cells, where they induce oxidative stress within mitochondria, impair electron transport chain function, and reduce ATP synthesis [31]. Additionally, the toxic environment created by the undegraded metabolites activates apoptotic signaling pathways, leading to programmed neuronal death [32]. The cumulative effects of these disruptions manifest as neurodegeneration and multi-system metabolic dysfunction.

The indispensable role of ECHS1 in BCAA metabolism underscores its potential as a therapeutic target. Furthermore, understanding how BCAA metabolic imbalances contribute to the pathogenesis of metabolic diseases offers new perspectives on the development of targeted therapeutic strategies.

### Mitochondrial function, oxidative stress, and apoptosis

Mitochondria serve as the powerhouse of the cell and play a pivotal role in maintaining redox homeostasis. ECHS1 is critical for sustaining normal mitochondrial function. When ECHS1 is deficient or dysfunctional, toxic metabolic intermediates, such as enoyl-CoA and partially metabolized fatty acids, accumulate within the cell. These byproducts interfere with the mitochondrial electron transport chain, leading to elevated ROS production. Excessive ROS attack mitochondrial lipids, proteins, and DNA, further impairing mitochondrial function and establishing a vicious cycle of oxidative damage [33]. Studies have demonstrated that mitochondrial oxidative stress is a major contributor to neurodegenerative diseases caused by ECHS1 deficiency, as neurons are particularly vulnerable to oxidative damage. This sensitivity is most pronounced in high-energy-demand regions such as the basal ganglia and cerebellum.

The disruption of mitochondrial function caused by ECHS1 dysfunction also triggers apoptotic signaling pathways. ATP depletion and elevated ROS levels activate apoptosis-related proteins, such as caspase-9, initiating the mitochondrial apoptotic cascade [34]. This effect is especially detrimental in the nervous system, where neurons rely heavily on energy supply and are highly susceptible to oxidative stress [35]. Loss of *ECHS1* function results in widespread neuronal apoptosis, which correlates with the pathological hallmarks of Leigh syndrome and ethylmalonic encephalopathy. Clinically, patients with ECHS1 deficiency often exhibit neurodegenerative symptoms, including abnormal muscle tone, delayed motor development, and cognitive impairments.

ECHS1 is implicated in various key molecular processes, including fatty acid β-oxidation, branched-chain amino acid metabolism, mitochondrial function regulation, oxidative stress balance, and potential signaling pathway modulation. Future research should focus on elucidating its specific molecular roles in these mechanisms, particularly its interactions with other metabolic pathways and enzymes. This will provide a comprehensive understanding of its role in metabolic diseases and pave the way for novel therapeutic approaches.

## Models for studying *ECHS1*

A thorough understanding of *ECHS1* gene function relies on robust experimental models. Through the construction of diverse mouse and cell models with *ECHS1* gene modifications, researchers have progressively elucidated its essential roles in metabolic regulation, mitochondrial function, and neuronal health. Research on *ECHS1* models not only deepens our understanding of disease mechanisms but also establishes a foundational basis for potential therapeutic interventions.

### Animal models

The *ECHS1* gene is pivotal in fatty acid β-oxidation and branched-chain amino acid metabolism, with deletion or mutation typically leading to lethal phenotypes at the embryonic stage in mice [4, 36]. This lethality is attributed to severe mitochondrial dysfunction, leading to an energy imbalance that compromises cellular and tissue survival. To circumvent the limitations of complete gene knockout, researchers have developed various animal and cellular models, including heterozygous knockout mice, conditional knockout mice, and iPSC models. These models serve as critical tools and provide foundational support for investigating *ECHS1*-related disease mechanisms.

#### Heterozygous *ECHS1* knockout mouse model

Mouse models provide a controlled in vivo environment for examining *ECHS1* functional deficiencies and their systemic impacts. Through the development of complete knockout, point mutation, and conditional knockout mouse models, researchers have comprehensively examined the *ECHS1* gene’s function across various physiological states and tissues. Because homozygous deletion of *ECHS1* generally results in embryonic or early postnatal lethality [4], researchers frequently employ heterozygous knockout mice (*ECHS1*^*+/−*^) to investigate its role in metabolic regulation. While heterozygous knockout mice exhibit near-normal survival and development, they display subtle metabolic abnormalities in fatty acid and branched-chain amino acid metabolism [31, 37]. These studies reveal partial mitochondrial dysfunction, particularly in high-energy demand tissues such as the liver and heart, suggesting that even partial *ECHS1* loss can profoundly affect metabolic homeostasis [37].

#### Conditional *ECHS1* knockout mouse model

To address the lethality associated with homozygous knockout, researchers have developed conditional *ECHS1* knockout mouse models to investigate gene function within specific tissues and developmental stages. Conditional knockout models allow precise *ECHS1* gene deletion in targeted organs or developmental periods, providing insights into the gene’s tissue-specific functions [38, 39]. For example, cardiac-specific *ECHS1* knockout results in myocardial hypertrophy and impaired heart function, underscoring the gene’s critical role in cardiac metabolism and muscle function maintenance [40]. Similarly, conditional knockout in the nervous system indicates that *ECHS1* is essential for neuronal energy metabolism and oxidative stress defense, with its absence leading to neurodegenerative changes and neuronal injury [41]. Additionally, temporally controlled knockout models have revealed *ECHS1*’s regulatory role in tissue maturation and metabolic homeostasis, providing valuable tools for examining its impact on aging and disease progression.

### Drosophila model (Drosophila melanogaster)

The Drosophila model is an invaluable tool for studying *ECHS1* mutations due to the high homology between the Drosophila and human genes. Recently, two novel *ECHS1* mutations (c.414 + 5G > A and c.310 C > G) associated with mitochondrial encephalopathy were identified in an infant, with in vitro studies showing these mutations lead to abnormal splicing and reduced enzyme activity [42]. Drosophila mutants effectively replicate the metabolic and neurological disruptions observed in human ECHS1 deficiency, providing a crucial platform for investigating ECHS1’s role in fatty acid β-oxidation and branched-chain amino acid metabolism, as well as advancing gene therapy research.

### Induced pluripotent stem cell model

With advances in iPSC technology, in vitro cell models have become invaluable for studying *ECHS1* mutations and related metabolic disorders. By generating iPSCs from patient somatic cells and differentiating them into specific cell types (e.g., neurons), researchers can recapitulate the impact of *ECHS1* mutations on cellular metabolism and mitochondrial function in vitro. Although *ECHS1*-specific phenotypes have yet to be confirmed in iPSCs, numerous laboratories are actively working with *ECHS1* iPSC models to better understand the gene’s role in mitochondrial function, energy metabolism, and oxidative stress. Currently, the primary application of iPSC models remains in recapitulating patient-specific cellular phenotypes, elucidating disease mechanisms, and conducting high-throughput drug screening, rather than direct therapeutic application.

In conclusion, diverse *ECHS1* mouse and cell models have progressively unveiled the gene’s importance in maintaining metabolic homeostasis, mitochondrial function, and neuronal health. These models provide scientific evidence for understanding the pathological mechanisms triggered by *ECHS1* mutations and establish a robust experimental foundation for gene editing and drug development.

## Therapeutic approaches for ECHS1 deficiency

### Dietary and pharmacological interventions

Dietary adjustments, such as restricting BCAA intake or implementing a low-protein diet, represent an effective strategy to reduce the accumulation of toxic metabolites [43]. Mutations in the *ECHS1* gene lead to metabolic.

disruptions in BCAA and fatty acid oxidation, resulting in metabolite buildup that impairs mitochondrial function [31]. By modifying dietary intake to limit these substrates, it is possible to mitigate some of the associated symptoms. For example, restricting BCAA intake helps reduce the accumulation of toxic metabolic intermediates, thus alleviating damage to the nervous and muscular systems. Additionally, the use of antioxidants, such as coenzyme Q10 and vitamin E, has shown potential in reducing oxidative stress and improving mitochondrial function, contributing to symptom improvement [44, 45]. Although dietary interventions and antioxidant therapies have shown positive effects in certain patients, responses vary significantly; hence, personalized nutritional management is crucial to maximizing symptom control and enhancing quality of life.

### Gene therapy

Gene therapy offers a potential curative approach for mutations in the *ECHS1* gene. In recent years, adeno-associated virus (AAV)-based gene therapies have demonstrated substantial therapeutic potential in animal models, with promising outcomes also observed in clinical settings [46–48]. For instance, delivery of the healthy RPE65 gene using AAV has led to significant vision improvements in patients with Leber congenital amaurosis [49, 50]. Similarly, AAV9-mediated delivery of the SMN1 gene has significantly improved motor function and extended survival in animals with spinal muscular atrophy [51–53]. These cases provide a theoretical basis for exploring AAV-based therapies for *ECHS1* deficiency.

In the context of *ECHS1* deficiency, AAV vectors can deliver the intact *ECHS1* gene to patient cells, thereby restoring enzyme activity. Serotypes such as AAV9 and AAVrh10, which efficiently cross the blood-brain barrier, have shown promise in targeting the nervous system in affected individuals [54–56]. However, gene therapy still faces challenges in clinical application, including immune responses and off-target effects, which require further investigation to ensure safety and long-term efficacy.

### Enzyme replacement therapy (ERT)

ERT aims to compensate for the functional enzyme deficiency caused by mutations in the *ECHS1* gene. By administering recombinant short-chain enoyl-CoA hydratase 1, ERT theoretically reduces toxic metabolite accumulation, leading to symptom improvement. This approach has made strides in other genetic enzyme deficiencies, such as Pompe and Fabry diseases, where regular enzyme supplementation significantly ameliorates clinical symptoms [57–61].

In the case of *ECHS1* deficiency, the primary challenge for ERT lies in ensuring that the supplemental enzyme can effectively enter mitochondria and remain stable at the target site. Researchers are currently investigating the use of mitochondrial targeting signal sequences to direct the enzyme into mitochondria, thereby enhancing therapeutic efficacy. In addition, modified recombinant enzymes are being developed to improve stability and extend half-life in vivo [62]. Although ERT has shown potential in preclinical studies, further validation is needed for its application in *ECHS1* deficiency patients.

### Extracellular vesicle (Exosome) therapy

Exosomes are nanovesicles that contain various bioactive molecules and have recently emerged as promising delivery tools [63]. Exosomes possess low immunogenicity and high targeting specificity and can be engineered to deliver functional genes, proteins, or drugs to specific cells and tissues [64]. Many studies prioritize enhancing delivery efficiency, targeting capabilities, and stability of therapeutic payloads instead of concentrating on immediate clinical applications [65, 66].

One notable advantage of exosome therapy is the ability to cross the blood-brain barrier, which is critical for addressing neurological damage in ECHS1 deficiency. Engineering mesenchymal stem cells (MSCs) to produce exosomes carrying ECHS1 mRNA or proteins may enable efficient targeted delivery to the nervous system. Studies have shown that MSC-derived exosomes exert neuroprotective effects in various neurodegenerative diseases, including mitigating oxidative stress and inflammatory responses [67, 68]. Although these methods have shown promise in preclinical studies, further optimization of exosome engineering processes and identification of the optimal routes and dosages are necessary. Currently, extracellular vesicle (EV)-based approaches are still in the preclinical stage, and their application in ECHS1-related disorders remains experimental. Most studies focus on improving delivery efficiency, targeting capability, and therapeutic payload stability, rather than immediate clinical application.

### Stem cell therapy

Stem cell therapy represents another potential therapeutic strategy for *ECHS1* deficiency, particularly in the context of neural repair [69]. By using autologous or allogeneic stem cells, such as MSCs or iPSCs, healthy cells can be generated in vivo to replace damaged neurons or other cell types [70]. Additionally, stem cells can secrete a range of growth factors and cytokines that promote tissue repair and regeneration [71].

In other metabolic and neurodegenerative diseases, stem cell therapy has shown some efficacy. For example, clinical studies using MSCs to treat Parkinson’s disease have observed improvements in motor function and reduced inflammatory responses [72, 73]. In *ECHS1* deficiency, stem cell therapy’s potential lies in providing a healthy metabolic environment to alleviate the metabolic burden and cellular damage caused by mutations. However, challenges remain, including the survival rate of transplanted cells, their ability to differentiate into specific cell types, and the potential risk of tumor formation. Current research on stem cell therapy for *ECHS1* deficiency is still in its early stages, yet it holds promise as a complementary therapeutic approach.

## Conclusion

The *ECHS1* gene plays a crucial role in cellular metabolism and mitochondrial function, particularly in fatty acid β-oxidation and branched-chain amino acid metabolism. However, despite the close association between *ECHS1* mutations and various metabolic disorders and neurological pathologies, there remain significant gaps in our understanding of its pathogenic mechanisms. Current research suggests that mitochondrial dysfunction and energy imbalance resulting from *ECHS1* deficiencies may underlie its pathogenicity, though specific molecular pathways and signaling processes involved require further investigation. For example, the precise functional changes caused by *ECHS1* mutations across different tissues and cell types remain unclear, particularly regarding their roles in neurological disorders.

Most existing *ECHS1* studies rely on animal models, primarily mice, which offer valuable insights into *ECHS1*’s metabolic functions across various tissues. For instance, heterozygous knockout mice have shown that partial *ECHS1* deficiency affects fatty acid and branched-chain amino acid metabolism, while conditional knockout models have elucidated its role in specific tissues, such as the heart and nervous system. Additionally, due to their genetic homology with humans, Drosophila models provide an efficient platform for investigating the effects of *ECHS1* mutations on metabolism and neural function. However, these models also have limitations. Homozygous knockout mice often exhibit early lethality, making it challenging to obtain comprehensive data on complete gene knockout, thereby limiting systematic research on *ECHS1* deficiency. Furthermore, despite extensive efforts to develop *ECHS1* iPSC models, no definitive *ECHS1*-specific phenotypes have been observed in iPSCs, which limits their application in cellular-level studies.

**Fig. 4:**
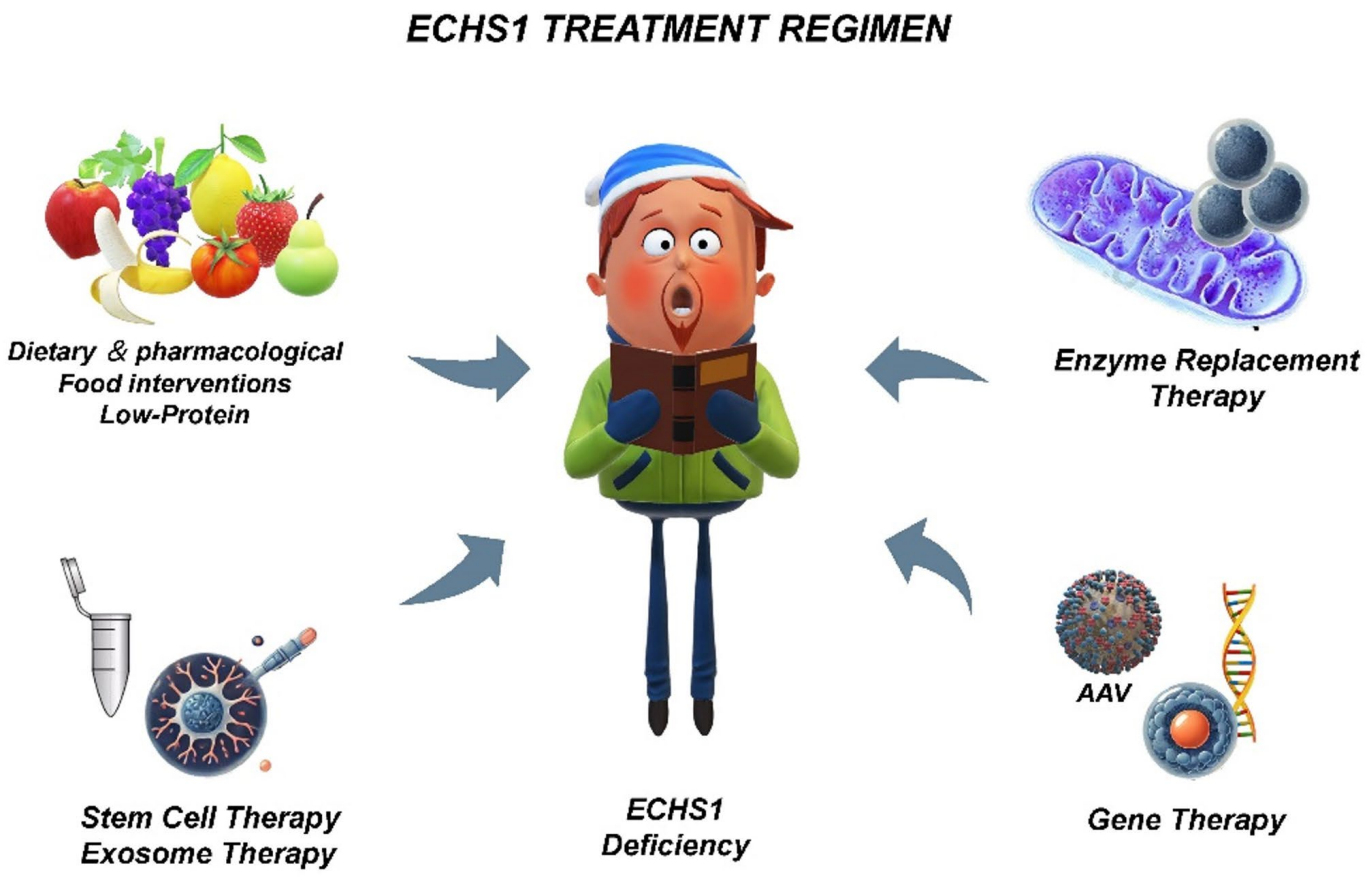
Potential therapeutic strategies for *ECHS1*-related disorders. This schematic outlines current and prospective treatment approaches for patients with *ECHS1* deficiency. Strategies include dietary and pharmacological interventions (e.g., high-calorie/low-protein diets), enzyme replacement therapy, gene therapy via adeno-associated virus (AAV), and cell-based approaches such as stem cell therapy and exosome therapy. Together, these modalities represent a multifaceted therapeutic framework targeting metabolic compensation, molecular correction, and regenerative repair

Future research should go beyond current animal models to develop models that more closely replicate human pathology, such as non-human primate models or advanced tissue-specific knockouts, for a more precise understanding of *ECHS1*’s role in disease. Additionally, research should integrate advanced technologies, such as gene editing and drug screening, to explore potential therapeutic strategies. With advances in CRISPR/Cas9 technology, preliminary results from gene repair targeting *ECHS1* mutations in mouse and iPSC models show potential for restoring mitochondrial function, suggesting the feasibility of personalized gene therapy. Furthermore, iPSC-based drug screening platforms may accelerate small molecule discovery, providing novel approaches for addressing *ECHS1*-related metabolic imbalances (Fig. 4).

While this review provides a comprehensive summary of current research progress on *ECHS1* models, it also has certain limitations. First, due to limited studies on the tissue-specific and developmental-stage-specific roles of ECHS1, discussions of ECHS1 functions across different tissues may not be exhaustive. Second, the lack of mature non-human primate models limits the availability of data closely resembling human pathology. Future research should build on existing models to address these gaps, thereby advancing our understanding of *ECHS1*’s pathogenic mechanisms and paving the way for effective therapeutic development.

## Data Availability

This article is a systematic review and does not involve the generation of any new datasets. All data supporting this review are obtained from previously published studies, which are appropriately cited within the manuscript.
